# Supplementary material for: Assessment of clinical outcomes with immune checkpoint inhibitor therapy in melanoma patients with CDKN2A and TP53 pathogenic mutations
Source: PLoS One. 2020 Mar 20;15(3):e0230306. doi: 10.1371/journal.pone.0230306 (PMC7083309; doi:10.1371/journal.pone.0230306)
Supplement: S1 Fig — (DOCX) [file pone.0230306.s001.docx]

Pathogenic mutation

Variant of undetermined significance

**Supplementary Figure 1:** Genotypes *CDKN2A*, *TP53* and quadruple wild type cohorts
