# Supplementary material for: Assessment of clinical outcomes with immune checkpoint inhibitor therapy in melanoma patients with CDKN2A and TP53 pathogenic mutations
Source: PLoS One. 2020 Mar 20;15(3):e0230306. doi: 10.1371/journal.pone.0230306 (PMC7083309; doi:10.1371/journal.pone.0230306)
Supplement: Attachment — Submitted filename: Response to Reviewers2.docx [file pone.0230306.s002.docx]

We thank you and the reviewers for the thorough evaluation of our manuscript and the thoughtful comments. In response to the reviewers’ comments, we are submitting a revised manuscript entitled “Assessment of Clinical Outcomes with Immune Checkpoint Inhibitor Therapy in Melanoma Patients with Somatic CDKN2A and TP53 pathogenic mutations.” We have made appropriate edits to the manuscript, which are highlighted, and address each comment point by point as below in bold.

1) Thank you for including your ethics statement on the online submission form: "This is a retrospective study which was approved by Mayo Clinic IRB(16-005168). No consent was needed as information was obtain anonymously. This study was conducted in accordance with principles for human experimentation as defined in the Declaration of Helsinki and International Conference on Harmonization Good Clinical Practice guidelines. No participating physicians have conflicts of interest to declare.". To help ensure that the wording of your manuscript is suitable for publication, would you please also add this statement at the beginning of the Methods section of your manuscript file.

**Addressed and added.**

2) In the ethics statement in the manuscript and in the online submission form, please provide additional information about the patient records used in your retrospective study. Specifically, please ensure that you have discussed whether the Mayo Clinic IRB specifically waived the requirement for informed consent since the data was analyzed anonymously. Thank you for your attention to this request.

**Addressed and Added.**

3) Please provide additional details in your Methods section regarding the methodology of the genomic profiling of the patients using the Solid Tumor Targeted Cancer Gene Panel.

**Expanded the methodology of NGS testing and this has been included under the methods**.

5) At this time, we ask that you please remove the word "trial" from the following sentence stated on Page 2 of the manuscript and in the Abstract:

'A total of 102 patients were included in this trial of which 14 had mutations of CDKN2A (CDKN2Amut), 21 had TP53 mutations (TP53mut), and 12 were QuadWT'

Please replace with the term 'retrospective study' to avoid potential confusion to readers that the manuscript describes the results of a clinical trial."

**Corrected and changed to study. Agree, the term study should be used.**
